# Engineering Cell Systems

**DOI:** 10.1155/2019/4685137

**Published:** 2019-06-02

**Authors:** Tiago G. Fernandes, Ricardo P. Baptista, Howard Kim

**Affiliations:** ^1^iBB-Institute for Bioengineering and Biosciences and Department of Bioengineering, Instituto Superior Técnico, Universidade de Lisboa, Av. Rovisco Pais, 1049-001 Lisbon, Portugal; ^2^The Discoveries Centre for Regenerative and Precision Medicine (Lisbon Campus), Instituto Superior Técnico, Universidade de Lisboa, Av. Rovisco Pais, 1049-001 Lisbon, Portugal; ^3^Cell and Gene Therapy Catapult, 12th floor Guy's Hospital, Great Maze Pond, London SE1 9RT, UK; ^4^The New York Stem Cell Foundation, New York, USA

The term “engineering cell systems” can be used to describe the application of engineering principles to the understanding of biological systems, solve biological problems, and ultimately contribute to the translation of new therapeutic approaches into clinical practice [1, 2]. Eventually, for the widespread use of stem cells in biomedical applications, it will be essential to recognize the complexity and the dynamics of stem cell systems, and therefore, the application of engineering principles will become crucial to the understanding of important biological questions, ranging from the cell level to the whole organism [3, 4]. This interesting concept is based on truly interdisciplinary methodologies and integrates contributions from multiple scientific and technological fields.

Currently, one can easily identify several key technologies that are gaining substantial attention in this area. Among these, we can find novel bioprocesses for the maintenance and expansion of human stem cells, as well as their differentiated progeny [5–7], and micro-/nanofabrication to produce tissue-like substitutes [8]. Moreover, these technologies should be developed with the objective of being implemented under Good Manufacturing Practice (GMP) conditions, in order to facilitate their translation to the clinic [9]. Several contributions featured in this special issue focus their attentions on these topics. Particularly, S. Dakhore et al. review current strategies for human pluripotent stem cell (hPSC) culture and discuss the challenges associated with the development of appropriate conditions to promote large-scale, quality-controlled expansion of hPSCs. On the same note, F. C. Paccola Mesquita et al. present an interesting study on the use of a closed hollow-fiber system that provides the necessary environment to scale up production of hPSCs while maintaining their stemness. They also demonstrate that laminin 521 can be used to promote the attachment of cells in the hollow-fiber reactor, resulting in a greater yield of viable hPSCs when compared with vitronectin. These results highlight the potential of such culture systems to yield high cell numbers in controlled environments, particularly when the scarcity of the initial cell population is an issue. Both contributions of A. T. Serra et al. and K. Chen et al. also tackle such questions by using microcarriers and spontaneously formed spheroids to expand cancer stem cells and compact bone-derived cells, respectively.

Another important objective of the field is the elucidation of the mechanisms that will allow the generation of functional human tissue-like substitutes [3, 6, 10]. To this end, mechanotransduction is of paramount importance, since this process may strongly influence cell fate and, thus, augment the precision of the differentiation of hPSCs into specific cell types, like cardiomyocytes. In this special issue, R. Santoro et al. review the main integrin-dependent mechanisms and signaling pathways involved in mechanotransduction, with particular emphasis in the cardiovascular field, focusing on biomaterial-based *in vitro* models of hPSC differentiation into cardiomyocytes. Generically, human morphogenesis is a complex process involving distinct microenvironmental and physical signals that are manipulated in space and time to give rise to complex tissues and organs. The development of organoids from hPSCs represents one reliable system to modeling such events, and T. P. Silva et al. review the main bioengineering methods used to promote the self-organization of stem cells, including assembly, patterning, and morphogenesis *in vitro*, contributing to tissue-like structure formation.

Other emerging topics of this area include the development of cellular products based on innovative scaffolds for the cultivation of stem/progenitor cells [11, 12] and controlled-release particles to program the differentiation of stem cells [13, 14]. In this special issue, M. Haagdorens et al. describe a collagen-like peptide biomaterial for tissue engineering of the cornea, while W. Qin et al. present a drug delivery system, consisting of calcium phosphate cement-containing chitosan with controlled release of metformin, to promote cell viability and odontogenic differentiation of human dental pulp cells, favoring dentin regeneration. In fact, stimuli-responsive materials, also known as smart materials, can change their structure and, consequently, original behavior in response to external or internal stimuli. D. K. Patel et al. also address this topic and review the physiochemical properties of graphene and graphene-based hybrid materials for stimuli-responsive drug delivery, tissue engineering, and antimicrobial applications. Additionally, taking advantage of the strong tropism that stem cells exhibit towards tumors, different researchers have proposed them as attractive candidates for targeted drug delivery in cancer treatment with minimal side effects. In this special issue, P. Wang and A. Aguirre describe the latest stem cell-based approaches for the treatment of cancer and also summarize the emerging imaging techniques being applied for monitoring anticancer stem cell therapy. This known tropism of certain stem cell populations to chronic tissue damage is typically complemented by regulatory effects on the immune microenvironment. Certain cells can regulate the immune microenvironment during tissue repair and provide a good “soil” for tissue regeneration. H. Li et al. discuss the regulation of immune cells by mesenchymal stem cells in the local tissue microenvironment and the subsequent tissue damage repair mechanisms.

Finally, the development of *in vitro* tests for toxicity, cell differentiation, genomic stability of expanded cells, and biocompatibility can profit from these scientific and technological advancements [4, 9, 15], and several contributions to this special issue focus on discussing these issues and the implications of these novel technologies for cell therapies, regeneration, and precision medicine.
